# Supplementary material for: Stress Responses to Hydrogen Peroxide and Hydric Stress-Related Acoustic Emissions (MHAF) in Capsicum annuum L. Applied in a Single or Combined Manner
Source: Plants (Basel). 2025 Aug 20;14(16):2591. doi: 10.3390/plants14162591 (PMC12389487; doi:10.3390/plants14162591)
Supplement: Supplementary file 1 [file plants-14-02591-s001.zip › plants-3793184-supplementary.pdf]

Supplementary material.

Table S1. Primers used for relative gene expression.

Primer name	Primer sequence 5′-3′
caMPK4-1-F	TTGACTTTGAGCAGCCATCTT
caMPK4-1-R	ACCATGAACTCGCCTCCGCTGCA
caMPK4-3-F	TGGTGCTAATGGAATGGTTTG
caMPK4-3-R	TTCTTTTGAGGTGGCCGTATA
caMPK6-1-F	AAAGCCTCTGTTTCCTGGTAG
caMPK6-1-R	CTCCTTCTGGGATCAAATGTC
caMPK6-2-F	CAGAGATCATGTACACCA
caMPK6-2-R	TCGCACCTGTTATTCTCCTTCTG
caMKK5-F	GATTTCATTGCCTGCTGTTTG
caMKK5-R	GTGCCTGATGGACCTGATTAC
Q-CaROS1-F	GGGGAAACTAACTATGTGC
Q-CaROS1-R	GATCTCCTCCTTAACCTCT
CaMET1-like1-F	CAGAAGCACAGGCAAATAGGAAATG
CaMET1-like1-R	CACTCCTAAATATGGGCGTACATGAAG
CaASR1-F	ACATGTCGGAGAACTCGGTG
CaASR1-R	TATCTTGTGCCTGTGTGCGT
CaNPR1-F	ACTTCTTCGCCGACGCCAAG
CaNPR1-R	GCCAACACATTCACCAGAGCATC
CaDEF1-F	GTGAGGAAGAAGTTTGAAAGAAAGTAC
CaDEF1-R	TGCACAGCACTATCATTGCATACAATTC
PR1a-F	GCCAAGCTATAACTACGCTAAC
PR1a-R	GCAAGAAATGAACCACCATCC
ERF1-F	CGCGTAATGGAATTAGGGTTTG
ERF1-R	CCTCATTGATAATGCGGCTTG
ACTIN-F	TGCAGGAATCCACGAGACTAC
ACTIN-R	TACCACCACTGAGCACAATGTT

A)

Post Hoc Comparisons - H2O2 ✻ MHAF

		Mean Difference	SE	df	t	Ptukey
0 0	1 0	-1.761×10^{-6}	2.306×10^{-4}	8	−0.008	.000
	0 1	-2.921×10^{-4}	2.306×10^{-4}	8	−1.267	.606
	1 1	4.897×10^{-4}	2.306×10^{-4}	8	2.124	.224
1 0	0 1	-2.904×10^{-4}	2.306×10^{-4}	8	−1.259	.610
	1 1	4.915×10^{-4}	2.306×10^{-4}	8	2.132	.222
0 1	1 1	7.819×10^{-4}	2.306×10^{-4}	8	3.391	.038*

B)

Post Hoc Comparisons - H2O2 ✻ MHAF

		Mean Difference	SE	df	t	Ptukey
0 0	1 0	−8.005	8.952	8	−0.894	.808
	0 1	−10.115	8.952	8	−1.130	.683
	1 1	35.563	8.952	8	3.973	.017*
1 0	0 1	−2.109	8.952	8	−0.236	.995
	1 1	43.569	8.952	8	4.867	.005**
0 1	1 1	45.678	8.952	8	5.102	.004**

C)

Post Hoc Comparisons - H2O2 ✻ MHAF

		Mean Difference	SE	df	t	Ptukey
0 0	1 0	−15.282	2.583	8	−5.918	.002**
	0 1	−16.511	2.583	8	−6.393	< .001***
	1 1	4.900	2.583	8	1.897	.301
1 0	0 1	−1.229	2.583	8	−0.476	.962
	1 1	20.183	2.583	8	7.815	< .001***
0 1	1 1	21.411	2.583	8	8.291	< .001***

D)

Post Hoc Comparisons - H2O2 ✻ MHAF

		Mean Difference	SE	df	t	Ptukey
0 0	1 0	−0.866	0.146	19	−5.918	< .001***
	0 1	−1.633	0.146	19	−11.157	< .001***
	1 1	0.617	0.128	19	4.810	< .001***
1 0	0 1	−0.767	0.172	19	−4.452	.001**
	1 1	1.483	0.157	19	9.437	< .001***
0 1	1 1	2.250	0.157	19	14.314	< .001***

E)

Post Hoc Comparisons - H2O2 ✻ MHAF

		Mean Difference	SE	df	t	Ptukey
0 0	1 0	0.043	0.004	16	10.809	< .001***
	0 1	0.054	0.004	16	13.413	< .001***
	1 1	−0.029	0.004	16	−7.296	< .001***
1 0	0 1	0.010	0.004	16	2.604	.081
	1 1	−0.073	0.004	16	−18.105	< .001***
0 1	1 1	−0.083	0.004	16	−20.709	< .001***

F)

Post Hoc Comparisons - H2O2 ✻ MHAF

		Mean Difference	SE	df	t	Ptukey
0 0	1 0	−1.194	1.460	19	−0.817	.845
	0 1	−3.182	1.665	19	−1.911	.257
	1 1	−21.627	1.665	19	−12.988	< .001***
1 0	0 1	−1.988	1.789	19	−1.112	.687
	1 1	−20.433	1.789	19	−11.424	< .001***
0 1	1 1	−18.445	1.959	19	−9.414	< .001***

G)

Post Hoc Comparisons - H2O2 ✻ MHAF

		Mean Difference	SE	df	t	Ptukey
0 0	1 0	0.685	0.499	20	1.373	.530
	0 1	−0.518	0.499	20	−1.037	.730
	1 1	−17.136	0.631	20	−27.150	< .001***
1 0	0 1	−1.202	0.547	20	−2.200	.157
	1 1	−17.821	0.669	20	−26.620	< .001***
0 1	1 1	−16.618	0.669	20	−24.824	< .001***

H)

Post Hoc Comparisons - H2O2 ✻ MHAF

		Mean Difference	SE	df	t	Ptukey
0 0	1 0	0.358	0.333	20	1.077	.707
	0 1	−0.321	0.333	20	−0.965	.771
	1 1	−7.504	0.421	20	−17.832	< .001***
1 0	0 1	−0.679	0.364	20	−1.864	.275
	1 1	−7.862	0.446	20	−17.615	< .001***
0 1	1 1	−7.183	0.446	20	−16.093	< .001***

I)

Post Hoc Comparisons - H2O2 ✻ MHAF

		Mean Difference	SE	df	t	Ptukey
0 0	1 0	0.648	0.293	19	2.210	.156
	0 1	−0.018	0.293	19	−0.060	.000
	1 1	−17.022	0.435	19	−39.150	< .001***
1 0	0 1	−0.665	0.321	19	−2.072	.198
	1 1	−17.669	0.454	19	−38.909	< .001***
0 1	1 1	−17.004	0.454	19	−37.444	< .001***

J)

Post Hoc Comparisons - H2O2 ✻ MHAF

		Mean Difference	SE	df	t	Ptukey
0 0	1 0	0.104	0.983	20	0.106	.000
	0 1	−3.585	0.983	20	−3.647	.008**
	1 1	−29.624	1.243	20	−23.824	< .001***
1 0	0 1	−3.690	1.077	20	−3.426	.013*
	1 1	−29.728	1.319	20	−22.541	< .001***
0 1	1 1	−26.038	1.319	20	−19.743	< .001***

Supplementary Figure S1. ANOVA post hoc comparisons. 00 stands for control group, 10 stands for H_2O_2 treatment, 01 stands for MHAF treatment, 11 stands for combined treatment. Antagonistic effect: A) Flavonoids, B) DPPH, C) ABTS, D) *def1*. Synergistic effect: E) SOD, F) *mkk5*, G) *mpk4-1*, H) *mpk6-2*, I) *ros1*, J) *met1*.

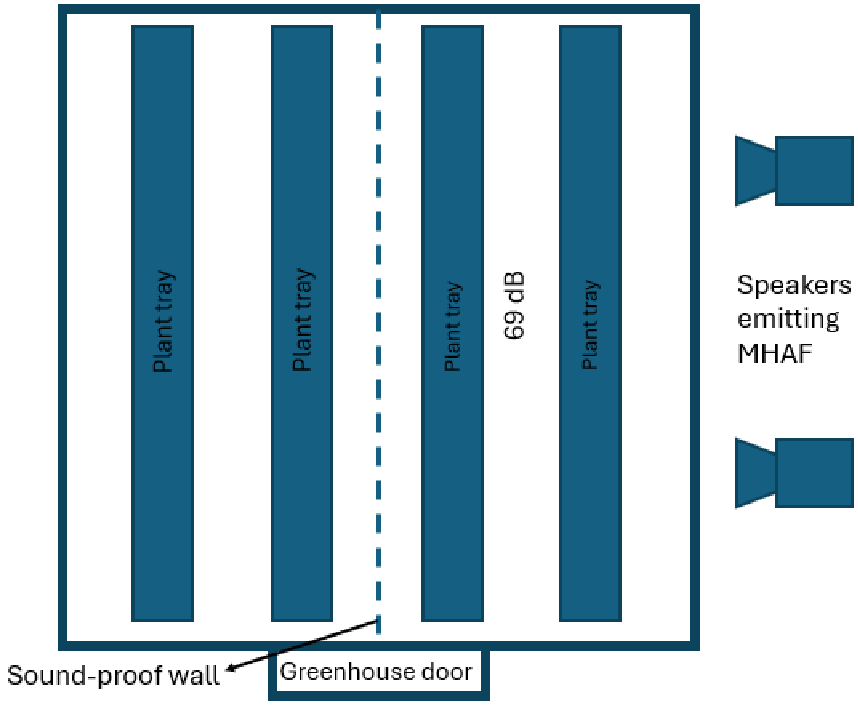

Supplementary Figure S2. Greenhouse adaptation used in the present study.
